# Supporting mental health conversations among youth with neurodevelopmental disorders through digital storytelling: A co‐produced study

**DOI:** 10.1002/jcv2.70179

**Published:** 2026-10-08

**Authors:** Patrick Jachyra, Yona Lunsky, Andrada Cretu, Elim Chan, Noah Barnett, Michael Barnett, Sheldon Gaboury, Cathy Gaboury, Austin Cosgrove, Susan Cosgrove, Claudine Aquino‐Evangelista, Carla Southward, Evdokia Anagnostou

**Affiliations:** ^1^ Department of Sport and Exercise Sciences Durham University Durham UK; ^2^ Azrieli Adult Neurodevelopmental Centre Campbell Family Mental Health Research Institute Centre for Addiction and Mental Health Toronto Ontario Canada; ^3^ Temerty Faculty of Medicine University of Toronto Toronto Ontario Canada; ^4^ Bloorview Research Institute Holland Bloorview Kids Rehabilitation Hospital Toronto Ontario Canada; ^5^ Province of Ontario Neurodevelopmental Disorders Network Toronto Ontario Canada; ^6^ Holland Bloorview Kids Rehabilitation Hospital Toronto Ontario Canada

**Keywords:** co‐production, digital storytelling, mental health, neurodevelopmental disorders, youth

## Abstract

**Background:**

Youth (aged 15–24 years) diagnosed with neurodevelopmental disorders (NDD) experience higher rates of co‐occurring mental health challenges and disparities in receiving timely care. A significant barrier to support is a lack of guidance for healthcare professionals (HCPs) on delivering conversations about co‐occurring mental health for youth with NDD. Limited guidance is amplified by a paucity of research which has included youths' perspectives on how to speak to them about co‐occurring mental health challenges. There is a need for research to include the voices of youth with NDD to ensure policies and practices reflect their lives, co‐occurring mental health needs and abilities.

**Methods:**

The study was co‐produced with a youth and parent advisory council. Nine youth with NDD, aged 16–21 (*M*_age_ = 18 years), created digital stories about their experiences with mental health support and how to foster conversations about co‐occurring mental health. The stories of six young men and three young women were analyzed thematically.

**Results:**

Negative experiences with support included feeling misbelieved, dismissed, and the use of esoteric medical/scientific language among HCPs. These experiences deterred future support seeking. A compassionate approach centered on rapport, trust, involving family members and establishing a space of psychological safety were foundational to support conversations about co‐occurring mental health. Explaining mental health challenges in an accessible way, tailored to their needs and involving youth in their care plan was essential to enable youth to feel safe and able to share their vulnerabilities.

**Conclusion:**

Encounters with HCPs can help, harm, and heal. Youth voiced the importance of HCPs playing a collective role in supporting mental health and shared approaches that were most supportive of their needs. A toolkit generated from digital stories to support conversations about mental health is offered. Digital storytelling can be a valuable and accessible methodology for youth with NDD with cathartic, methodological and empirical benefits.

## Key Points

**What's Known?**

- Youth with neurodevelopmental disorders (NDD) experience high rates of co‐occurring mental health challenges and experience disparities receiving support. There is a lack of knowledge and tools for healthcare professionals (HCPs) to support discussions about mental health.

**What's New?**

- Youth with NDD shared how HCPs can support conversations about mental health through digital storytelling.
- Negative experiences included feeling misbelieved/dismissed, which deterred future support seeking. Positive experiences included personalization of challenges, establishing rapport, a space with psychological safety, including family, and a collective role for HCPs to foster discussions about mental health.

**What's Relevant?**

- Compassionate and relational tools are offered to support HCPs to deliver accessible conversations about mental health for youth with NDD.

## INTRODUCTION

Youth (aged 15–24) diagnosed with neurodevelopmental disorders^1^ (NDD) such as autism spectrum disorder, attention deficit hyperactivity disorder, obsessive‐compulsive disorder (among others) often experience co‐occurring mental health challenges (Boulton et al., 2023). Compared to their age‐related peers, youth with NDD experience co‐occurring mental health challenges at higher rates (Augustine et al., 2022), with anxiety (Lecavalier et al., 2019) and depression (Rosen et al., 2018) being the most common. Although the etiology of co‐occurring mental health challenges in youth with NDD is emerging, the combination of genetic, environmental and stress related factors (Homberg et al., 2016; Zhou et al., 2026) have been associated with higher rates. Social and systemic issues such as stigma/misconceptions about NDD (Taylor et al., 2026), diagnostic overshadowing (mental health challenges are attributed to NDD) (Ashworth et al., 2025) and poor fit between health/social services (Ashworth et al., 2025) and individual needs (Jachyra et al., 2022) can contribute to co‐occurring mental health challenges. Unsupported co‐occurring mental health challenges and their sequalae are concerning as they can interfere with activities of daily living, difficulties in educational attainment (Dalsgaard et al., 2020), decreased opportunities for employment (Toft et al., 2021) and negatively impact quality of life (Augustine et al., 2022). Given the aforementioned impacts of co‐occurring mental health challenges, supporting the mental health of youth with NDD has been identified as a global priority by self‐advocate (Autistica, 2024), government (Public Health Agency of Canada, 2024) and interest‐holder groups (Healthy Minds, 2024) so that youth with NDD can flourish to their full potential.

Although the mental health of youth with NDD is recognized as a priority, evidence suggests that youth experience significant barriers accessing and receiving timely and effective support (Weitzman et al., 2024). At a systems level, accessing mental health support often is prohibitive where referrals, waiting times (McKenna et al., 2024) and an NDD diagnosis can preclude access (Ashworth et al., 2025) to mental health support. These barriers are amplified when transitioning into adulthood (Eklund et al., 2016) where a discontinuity of care and delays in support can exacerbate distress and negatively impact wellbeing (Eklund et al., 2018). At the individual level, barriers among youth such as motivation to engage in self‐management strategies (Bussing et al., 2016), low desire for support, belief that no change is needed (Sibley et al., 2023) and limited parental involvement can shape seeking and engaging in mental health support (Sibley et al., 2023). Finally, a lack of training among healthcare professionals (HCPs), limited confidence in supporting youth with NDD (Ashworth et al., 2025) and a lack of tools to guide practice (LaPoint et al., 2024) can contribute negative experiences with mental health support (Crane et al., 2019). Given these manifold barriers, mental health difficulties often go untreated (Anderson & Butt, 2018) for youth with NDD, contributing to unmet needs and compounding health disparities. There is a need for research to examine how to mitigate these barriers and improve support for co‐occurring mental health needs.

To date, research has predominantly been conducted with parents/caregivers, HCPs or included youth with other adult stakeholders (LaPoint et al., 2024). Despite calls to include the voices of youth with NDD in mental health research (Sibley et al., 2023), little is known about youths' preferences when having conversations about mental health with HCPs, and their experiences receiving support for co‐occurring mental health challenges. The lack of research presents a knowledge and practice gap as there is little guidance on how to deliver mental health conversations among youth with NDD from their perspective. Including youth with NDD in research about their experiences of mental health support is vital to ensure they have voice in shaping policy and practice which directly impact their lives. Learning from their support experiences and including their voices has the potential to generate conversations about their mental health which better reflect their diverse needs, interests and abilities, which currently are limited. Generating practices which reflect their lives is essential, as NDD and co‐occurring mental health historically have been researched and treated separately in healthcare systems. This can present challenges in understanding and supporting the presentation(s) of NDD and co‐occurring mental health challenges and how to deliver personalized support (LaPoint et al., 2024) as mental health assessment, strategies and interventions do not “typically take into account neuro‐developmental differences, such as the potentially unique needs of autistic children and young people (Ashworth et al., 2025, p. 2112). As a result of this underlying dualism, there is a need for transdiagnostic strategies to support co‐occurring mental health challenges which may be amenable across NDD as youth often experience co‐occurring NDDs (Azim et al., 2025), have frequent encounters with healthcare systems (Leyenaar et al., 2024) and experience barriers to support because of co‐occurring conditions (Babalola et al., 2025). Knowledge and practice gaps about youth's preferences, voices and experiences with co‐occurring mental health support prompted the need for this study to better support the mental health of youth with NDD. The purpose of this study therefore was to: Explore youth's experiences receiving support for co‐occurring mental health challenges; and examine how HCPs can foster and deliver supportive mental health conversations from the perspectives of youth.

### Co‐production

This study was conceptualized by members of a youth program advisory council (PAC) and a parent PAC who are part of the Province of Ontario Neurodevelopmental Disorders Network (POND). This research network is participant‐driven, seeking to better support the lives of people with NDD and those who care for them through research. Through a priority setting exercise, 10 priorities were derived from 1200 questions, across 300 participants in Ontario and developed through engagement with individuals with lived experience, families, friends, carers and HCPs (POND, 2026). Three members of the youth PAC and four parents brought the need for the research and partnered with researchers to form a team. Youth members all had at least one NDD diagnosis, and parent members all cared for a child diagnosed with an NDD. Co‐production was critical to ensure that the research meaningfully reflects and supports youth with NDD, those who care for them, and their experiences of healthcare (See Table S1). An equitable approach to co‐production was taken where youth and parents were equal partners with planning, power and had decision‐making responsibilities (Tan et al., 2025). As such, they were integral team members developing study design, grant writing, data analysis, and manuscript writing. They rightly are authors of this paper. From the outset of the study, the team recognized the presence and impact of visible and invisible power dynamics and sought to work through these through open and on‐going discussions about power to create a safe space. The team sought to build rapport through regular meetings, empowerment through valuing differing perspectives and providing voice, choice and decision making, particularly for youth team members. The team met regularly between 2020 and 2025 guided by the needs of research, with variance between bi‐weekly, monthly and quarterly meetings.

## METHODS

Digital storytelling (DST) was identified as an optimal method to engage youth with NDD as it can be a powerful medium to provide participants with a mode of expression about difficult and sensitive topics such as co‐occurring mental health challenges. Through a structured workshop process, DST is an arts‐based methodology combining photos, videos, audio narration, music, artwork, text and drawings to create short, evocative and engaging audio‐visual vignettes or digital stories (Gladstone & Stalius, 2019). Digital stories are highly personal and authentic autobiographic accounts of the lives of ordinary, often marginalized individuals (Gladstone & Stalius, 2019) and have been used in research detailing experiences of psychosis (Boydell et al., 2017), exclusion among autistic youth (Jachyra et al., 2021) and autistic youth transitioning to adulthood (Lewis‐Dagnell et al., 2024). We selected DST for four reasons. First, we sought to provide youth with a mode of expression that creatively captured their experiences and conversations about a difficult topic. Second, we sought to support diverse communication needs and abilities of youth with NDD, particularly youth who communicate in ways other than verbal speech. The flexible nature of DST enabled participants to use different mediums of communication to share their stories and work at their own pace. Third, we sought to provide participants with new skills (the process of capturing and creating content) and tangible outcomes (a digital story), as an effort in reciprocity. Fourth, DST generated a form of analyzable data and a knowledge mobilization tool given the accessible and impactful nature of firsthand accounts. We selected DST with the aim of sharing digital stories with HCPs and draw on the stories to generate neuro‐affirming toolkits to improve clinical encounters. Shaping healthcare practice was important as DST has been used previously to humanize healthcare care (Benham‐asl et al., 2024) and improve knowledge and attitudes about the complexities of social determinants of health among HCPs (Maragh‐Bass et al., 2025).

### Procedure

Youth with NDD were recruited from the province wide POND network. In this research network, participants complete baseline and follow‐up measures over time and are invited to participate in new research studies. Youth who met the following criteria were invited to participate: Clinically diagnosed with an NDD; Aged 16–21 years; Experienced co‐occurring mental health symptoms or a co‐occurring mental health diagnosis; Received support from a licensed and regulated health professional for a co‐occurring mental health concern(s); Lived in Ontario; Able to provide independent consent; Proficient in English; Access to a computer. The upper end of adolescence was selected as youth with NDD can access certain pediatric supports until the age of 21 in Ontario, Canada. During participant screening, youth were asked to confirm eligibility criteria, including whether they had received support for a mental health concern from an HCP. As illustrated in Table 1, nine youth consented to participate in the study. Eight youth primarily received support as outpatients, across primary, and secondary care. In this study, primary care includes support in the community with general practitioners and allied health professionals such as social work, psychologists, and naturopathy. Secondary care involves specialist care such as emergency medicine or referral‐based care with neurologists, psychologists and psychiatrists. Two youth completed an inpatient program, with one participant drawing on their experiences as an outpatient in their digital story. Participants engaged in seven workshops (see Table 2) during May–July 2021 which were co‐produced by the research team and the expert DST community partner. The workshops were conducted virtually due to the COVID‐19 pandemic.

**TABLE 1:** Participant demographics.

Demographic information	Number and percentage (, %)
Age in years	
16	1, 11
17	2, 22
18	3, 33
19	2, 22
21	1, 11
Gender	
Young men	6, 66
Young women	3, 33
Primary neurodevelopmental diagnoses	
ASD	2, 22
ADHD	1, 11
ASD and ADHD	3, 33
Obsessive compulsive disorder	1, 11
Tourette's syndrome	1, 11
Learning disability	1, 11
Co‐occurring diagnoses	
General anxiety disorder	3, 33
Anemia of chronic diseases	1, 11
Experiences of mental health symptoms or behaviors	
Low mood and sadness	3, 33
Chronic stress	3, 33
Feeling anxious	6, 66
Worry	4, 44
Mood swings	2, 22
Panic	4, 44
Suicidality	2, 22
Self‐harm	3, 33
Intense anger and irritability	3, 33
Negative and intrusive thoughts	2, 22
Withdrawal	2, 22
Phobia	1, 11
Type of healthcare received	
Primary care	3
Secondary care	6
Region	
Southern Ontario and Greater Toronto Area	7, 78
Northern Ontario	1, 11
Eastern Ontario	1, 11

Abbreviations: ADHD, attention deficit hyperactivity disorder; ASD, autism spectrum disorder.

**TABLE 2:** Workshop plan.

Workshop 1	Introduce project, storytelling circle
Workshop 2	Script writing, selecting images, and creating a story board
Workshop 3	Final script edits, final storyboarding
Workshop 4	Voice recording
Workshop 5	Video editing part 1‐basic editing
Workshop 6	Video editing part 2‐special effects
Workshop 7	Final edits, screening of digital stories and celebration

Each workshop was 3.5 h in duration, held on consecutive weekends. The morning group had five participants, and the afternoon group had four participants. Youth participants selected the group that suited them best and remained in that group to build rapport. Two DST staff and two research team members attended all workshops to support participants. Group discussions and brainstorming occurred in the main virtual room and breakout rooms were used for independent digital story work, with facilitators available to support. To initiate discussion with youth, prompts were used such as: Can you share a time you needed help with your mental health and it felt supportive, what made it supportive and worked well; Can you share a time you needed help with your mental health and it did not feel like a positive experience, what did not work well; What could HCPs do to make discussions about mental health more supportive? Structured breaks were provided and youth were also encouraged to step away from the workshop at any time given the sensitivity of the research and labor‐intensive process of creating a digital story. At the end of the 7th workshop, a storytelling circle was held, where individual digital stories were screened with participants from both workshop groups, along with parents/caregivers. Each youth received a copy of their digital story and gift card for their contributions. The study was approved by the Holland Bloorview Kids Rehabilitation Hospital (REB 0330‐2021) and the Centre for Addiction and Mental Health (REB 133‐2020) research ethics committee.

### Analysis

Digital stories were analyzed thematically using a flexible coding and memoing approach guided by Miles and Huberman (1994). Grounded in interpretivism, analysis commenced by transcribing digital stories verbatim, noting the tone of voice, tone of language and nature of interactions shared by participants due to the sensitive topic under study. Interpretivism was selected as it aligned onto‐epistemology of the study objectives, and the use of DST where we approached mental health “as a dynamic, socially embedded phenomenon rather than a fixed diagnostic category” (Beg, 2025). Three team analysis meetings were held, and each meeting was 90–120 min in duration. Each transcript was coded by three members of the research team (P.J., A.C., E.C.) who had academic training in qualitative analysis and disciplinary training spanning life sciences, psychology, neuroscience, rehabilitation sciences and public health. At the first meeting, analysis training was provided for all team members, where a transcript was analyzed as a team to practice coding. Analysis continued by discussing each transcript where codes were identified, added, discussed and added into a code book. Codes were compared across transcripts and refined through iterative feedback. Initial coding explored experiences with co‐occurring mental health challenges, experiences seeking support and the processes which shaped interactions during support seeking. At the end of the first meeting, analytic memos were written to further conceptualize and reflect on generated codes and shared with the entire team to think through what was seen and potentially unseen in the data. The second meeting combined refined codes into categories. A cross‐analysis across the transcripts was completed to examine relationships, patterns across participants and divergences. Analytic memos and iterative group discussions further refined categories and candidate themes. The final meeting focused on generating themes from categories and analysis continued through group discussion until final themes were established. Throughout analysis, the team engaged reflexively through iterative data analysis, group discussions and peer debriefing led by the first author who had extensive experience conducting qualitative research and co‐production among youth with NDD. Engaging in reflexivity was critical, as our insider roles were a key resource to develop an in‐depth understanding of mental health experiences among youth with NDD. Concomitantly, it was important to reflect how our individual, and collective identities, life experiences, researcher and clinical training shaped analytic choices as our team included: experts through experience which included youth and parents; clinicians; a knowledge translation specialist; and researchers with expertise in DST, psychology, mental health, pediatric medicine, neurology, qualitative research and co‐production.

## RESULTS

### Impact of negative healthcare interactions

Across the digital stories, 6 out of 9 (67%), youth reported negative experiences with HCPs whilst seeking mental health support. These experiences occurred in both primary and secondary care settings and included being treated condescendingly, the use of esoteric medical and scientific language, HCPs talking over them and their families and or speaking harshly. During these interactions, youth highlighted they did not feel heard and that assumptions were made about how they were feeling and the support that was needed. These negative experiences were detrimental as they made youth feel shamed, embarrassed, and abnormal. It also exacerbated the challenges they were experiencing when requiring support.

He started talking off saying I was just doing this for attention (attending the emergency room while being in crisis). He wouldn't listen to what I was saying. He was talking over me. He talked to my mom in a different room. He spoke to her really harshly. We were expecting to be taken seriously.

(Story 1)

Once I was told he helps children and teens which other doctors reject and neglect to help because they are too complicated.

(Story 2)

Medical practitioners would speak to Wormy using medical and scientific words that he could not understand.

(Story 3)

I was made to feel I was not normal, whatever that is.

(Story 7, in‐patient)

#### Personalization and mistrust

During interactions with HCPs, a lack of personalization of mental health challenges was noted. Youth reported some HCPs did not take the time or seemed to express interest in understanding their individual needs, develop an appreciation for the challenges they were experiencing and minimized what they were going through during a time need. Interactions which lacked personalization made it difficult for youth to trust HCPs and made it difficult to work through what they were experiencing as they did feel valued or understood. These interactions were a barrier to build rapport, empathetic understandings and were isolating when youth sought support:

Help us through it, ask us questions. Ask how we’re doing. Don’t let us cry alone because that makes us feel worse. You only cry for help, if you believe there is help to cry for.

(Story 7, in‐patient)

I thought doctors should not be trusted.

(Story 2)

In the end, I did a voluntary treatment program. I was in a psych ward for seven weeks. It was the hardest time of my life. I was locked up in a place because I was a danger to myself…this could have been avoided and I could have been an outpatient.

(Story 1)

Negative interactions built mistrust. Yet trust was a foundational element needed to initiate conversations. Mistrust of HCPs decreased the predilection of seeking help and some youth stopped seeking help altogether due to the negative interactions they experienced. The importance of personalization, rapport, and a safe space where youth could share vulnerable aspects of their lives was critical. Highlighting their experiences and impacts of mistrust, youth noted:

They made me scared to seek medical help. At this point, we were trying to leave with whatever dignity we had.

(Story 1)

Wormy did not always trust Dr. Heartstein.

(Story 3)

I felt like I couldn't trust anyone because if I'd tell something they'd make me stay there longer. I didn't really like the people working there because they seemed like they were trying to force us to get better by using medication. I had to do what they asked. I felt like they were experimenting on us to see if they could fix our emotions.

(Story 7, in‐patient)

Negative interactions experienced by youth are noteworthy as stress from healthcare settings impacted daily life such as relationships with other adult figures. For example, one participant described how their schoolteacher did not understand their non‐verbal communication or have context for the changes in their emotional states and behavioral expression. This similar experience with their teacher triggered flashbacks to negative experiences with HCPs which had damaging impacts as it contributed to fear and exclusion in school:

It got to the point that every time the specialists touched my body I was scared. In my mind, I thought that doctors should not be trusted. By the time I was 10, I was so stressed that I was shouting out in class, and I had to leave the classroom and go for walks all the time to calm down. Many teachers did not understand my nonverbal communication and didn't include me in the class activities.

(Story 2)

### Mental health support as a collective responsibility for HCPs

Youth received support from diverse HCPs such as a psychologist, psychiatrist, physician, social worker, neurologist and a naturopath. The diversity of HCPs highlights the frequent contact youth have with the healthcare system for varying, mental, social and physical health needs, where co‐occurring mental health challenges were identified. Across interactions, HCPs who took the time to explain the mental health challenges youth were experiencing in an accessible way and guided youth how to use self‐management tools in their daily lives enabled youth to engage with support that was offered. Developing shared understandings of co‐occurring mental health mattered as it allowed the HCP and youth to create a shared understanding of what they were experiencing. When a shared understanding was achieved, youth and HCPs co‐created approaches to the self‐management of symptoms, especially when there were multiple or competing needs. This was important as mental health challenges were experienced at different frequencies, intensities, types, and at different times. The power of shared understandings and impact of receiving support tailored to individual needs is highlighted below in Table 3.

**TABLE 3:** Impact of support across HCPs.

Story number	Supporting quote
Story 6	*My helpers taught me how to be self‐confident in my abilities to fight my battles, thoughts and how to defy my fears all through visualization. Of course, I am still learning how to get the full scope of knowledge; the full toolbelt of skills to use to my advantage*.
Story 8	*My parents took me to see a psychologist. She gave us some strategies to work with. She taught me breathing exercises like “box breathing” and “deep breathing” and how to talk to people I trust and get along with. We also talked about how listening to music makes me feel better. She taught me how to self‐advocate and how to talk to someone I trust if something ever happened again. The psychologist was helpful because she spoke to me in an easy language that I could understand and taught me a few exercises that I could do by myself when I'm stressed*.
Story 3	*But Dr. Heartstein took his time and reassured Wormy that it would not hurt. Wormy actually found the acupunctures helpful. He also introduced Wormy to meditation and breathing techniques which would help Wormy stay calm. Wormy took the suggestion and began drawing out his emotions and using meditation as a way to help with his anxiety. The night before his exam, Wormy began to draw and he played the meditation music to relax his nerves*.
Story 9	*Eventually with a lot of support and practice from social workers, Matthew will learn strategies and skills and be able to calm down on this own*.

Abbreviation: HCPS, healthcare professionals.

A collective opportunity for HCPs (within their capacities) to support youth with NDD was identified. The quotes above highlight how an individualized and needs‐based approach allowed youth to draw on different tools at different times for their varying needs from diverse HCPs across diverse healthcare settings where co‐occurring mental health challenges emerged as a presenting problem. Whereas some youth were seen by mental health specialists, other youth reported positive experiences with HCPs who were non‐specialists but employed a co‐created and tailored approach for support. This approach enabled youth to compartmentalize some of the mental health difficulties they were experiencing and guiding them toward a toolbox of support.

### Relational aspects of mental health discussions

Across eight of the nine digital stories, supportive mental health care was experienced when conversations and support were tailored to the strengths and abilities of youth with NDD. Personalized support which reflected their needs, allowed youth to feel valued and eased them into conversations about their mental health.

And also, he asked, like, straightforward literal questions because I took things literally. And it made things easy to understand like what he was asking.

(Story 5)

They gave me guidance to allow me to live my best life.

(Story 2)

#### Clear communication as a foundation for optimal support

The use of direct language and relatable examples helped youth comprehend the co‐occurring mental health challenges they were experiencing and identify avenues for support in primary and secondary care settings. The importance of communicating in direct and relatable ways was expressed by a youth who noted that their general practitioner helped them understand mental health by comparing it with a topic that was relatable to them (e.g., a video game). When esoteric language was used, youth felt excluded as it made them feel that their lived experience was not taken seriously and were infantilized. Language used by HCPs that was too abstract created challenges in processing and actioning the support provided as it did not seem like HCPs understood their individual needs or that communication and processing differences may exist among youth with NDD. As highlighted in Table 4, direct conversations with clear language which matched an individual's level of understanding allowed youth to understand, communicate effectively and implement the support that was provided by HCPs.

**TABLE 4:** Importance of communication.

Story number	Supporting quote
Story 4	*One day I saw a neurologist. She took the time to get to know me and my interests, like planes and trains. She never rushes me and really listens to me, and she doesn’t talk to me like I’m a little kid. This makes me feel important and wanted. It helps me to tell her all the hard things that I struggle with like my freakouts. She suggested that I should let it take its course, but in a safe place like my bedroom. I started doing it. I would calm down quicker and then I would come out of my room feeling better*.
Story 5	*Uh the doctor helped me try to, like, understand what was stuck…by comparing it to how online video games work: where sometimes the server can completely shut down because there’s too much information coming into the network. And there's no control. And well sometimes they crash. You can't get them back. Uh, the doctor did explain that there's lots of things I can do to try and like help with this interior world…Like do exercise or meditation techniques*.
Story 9	*The idea that Matthew should forgive and forget was too abstract for him. What helped him the most was clear, direct and concise language*.

#### Trust as a prerequisite for disclosure

The elements described above created space for building therapeutic rapport and support. These interactions were critical to build trust with the HCP as the challenges youth were experiencing were perceived as scary, overwhelming and at times felt like they were out of control. Trust was developed through sustained and repeated positive interactions and built when one's individuality was acknowledged. Trust was also built when youth felt treated with respect and dignity, and when they felt seen as a person, rather than their specific diagnostic label. The impact of positive interactions was critical to provide appropriate support, facilitate resilience and facilitate patient growth. This was explained by a youth below:

He didn’t assume that I couldn’t understand. And he actually, like, saw me as a person, not just a diagnosis. This sort of, like, casual sort of appointment was really important for, like, building trust, especially when I needed help.

(Story 5)

A trusting relationship with the HCP allowed youth to open‐up about their challenges and made them feel safe to allow HCPs to provide suitable support. Repeated positive encounters with HCPs not only built trust but also enabled youth to engage in learning strategies to self‐manage their symptoms. This strengths‐based approach facilitated growth as highlighted in Table 5 below.

**TABLE 5:** Impact of trusting relationships.

Story number	Supporting quote
Story 2	*Finally, I was referred to a neurologist…. When we first met, right away he said “we are going to take care of you”. He encouraged me to use my phone to communicate instead of just talking to my parents. He used a private room where I could express myself instead of the open concept rooms at the hospital. He was the first person who asked me what I thought I needed. He made me feel included with the medical decisions that were being made. He made me feel safe again. My neurologist also found the doctor who gave me the right diagnosis, Tourette's. This radically changed the way that I saw things. I wasn’t going to die, and I learned to self‐manage my symptoms…Now I am a student in Indigenous Studies at Trent University. I have grown personally, spiritually and academically and want to go into Indigenous law.*
Story 1	*Now I am 16 and have a proper diagnosis and a trusted doctor. I'm on proper medication and I have amazing people who I've built relationships with. The hospital I go to now helps us connect with different programs and resources.*

### Compassionate dimensions of mental health care and youth voice

The tone, message and the specific approach used to deliver information by HCPs for youth and their families was foundational. Including parents/caregivers as part of the process also was important as they played a pivotal role in the daily life of youth and their mental health needs beyond the clinical encounter. This was important as parents/caregivers can provide valuable insights into the specific needs and lives of youth with NDD. Yet, it was not always routine practice for parents/caregivers to be included during clinical encounters with HCPs. When youth had their parents/caregivers included as part of collaborative decision‐making process for their treatment plans, youth shared that it enabled them to feel respected, reassured, and at ease. This mattered as they felt supported and opened avenues for hope and recovery as highlighted below.

He used a private room where I could express myself instead of the open concept rooms at the hospital. He was the first person who asked me what I thought I needed. He made me feel included with the medical decisions that were being made. He made me feel safe again.

(Story 2)

They should treat us like we're human beings not animals. We're smarter than people think we are. They should try to talk to us, support us. I like how being included is making decisions for my care plan. Don't yell at us or ignore us.

(Story 7 in‐patient)

Youth highlighted how being cognizant of delivery and the impact of the interactions can impact the success of future healthcare interactions and seeking help in times of need. A needs‐based approach where the strengths and challenges of youth with NDD are recognized, was critical in delivering supportive conversations about mental health. This was articulated consistently across the stories and poignantly by two youth highlighting how to communicate with youth with NDD about co‐occurring mental health challenges.

Medical professionals need to understand that what you say to patients can stick to them forever.

(Story 1)

Dr. Heartstein listened to Wormy and spoke to him slowly and that helped him stay calm.

(Story 3)

Youth identified the importance of being treated with dignity and compassion and the importance of being included in conversations about their health. Including youth in discussions about their mental health and avenues for support in an accessible way built a sense of mutual respect and understanding. A compassionate approach mattered as it made them feel that they deserve help, despite previous negative healthcare experiences. When these elements were delivered by HCPs and aligned with their individual needs, it set youth up for success during mental health conversations:

It helped me feel more valued and understood.

(Story 5)

We all deserve to be treated with compassion, respect and to be included in conversations influencing our health.

(Story 2)

People need to understand we deserve help. I'm sure there are people who went through similar things as me.

(Story 1)

## DISCUSSION

This study examined youth's experiences with support for co‐occurring mental health challenges and examined how HCPs can deliver supportive mental health conversations for youth with NDD. This paper makes a novel and original contribution to the knowledge base with the use of DST, co‐production and the first paper in Canada to directly include the perspectives of youth with NDD. Given that clinical encounters have the potential to help, harm, and heal, study findings have implications for inpatient and outpatient care as the challenges and positive experiences identified can have cross‐cutting impacts across all levels of healthcare. As shown in the results, negative experiences occurred when youth were misunderstood/minimized and or a misalignment between individual needs and the support that was offered. Negative experiences with mental health support in this study are consistent with previous research where youth with NDD experience barriers accessing and receiving mental health support in a neurotypical world (Crane et al., 2019). Negative experiences matter, as they not only contribute to unmet needs but also compound health disparities as our findings showed that they can dissuade future mental health support. While youth in the general population also report negative experiences (Persson et al., 2016), youth with NDD are uniquely vulnerable due stigma/misconceptions (Taylor et al., 2026), diagnostic overshadowing, and being turned away from specialized mental health support due to an NDD diagnosis (Ashworth et al., 2025). These unique challenges have been noted among autistic young adults where autistic voices often are excluded during healthcare encounters as power tends to be concentrated in the hands of HCPs and hierarchies of knowledge (Uisma et al., 2026). As such, certain kinds of knowledge and knowers often are excluded during healthcare interactions as reductive assumptions about NDD are embedded in diagnostic and practices, which in turn do not account for the diversity of expression and experience of the world (Uisma et al., 2026). A lack of representativeness of how NDD is understood, conceptualized and societally valued in turn perpetuates neuro‐normative assumptions (Jachyra & Townsend, 2026) which take for granted what “typical” or “normal” presentations of mental health and behavior may look like. This in turn can overlook the specific needs of youth with NDD, particularly in a mental health context. Given the impacts of negative interactions described in this paper, a toolkit was co‐designed with youth with NDD, parents, researchers and clinicians to support HCPs and youth with NDD (see ClinicianToolkit). Informed by the digital stories from this study (DigitalStories), five relational and strengths‐based strategies are offered with DST excerpts to provide HCPs with strategies to create positive encounters and support the diverse needs, interests and abilities of youth with NDD. The toolkit provides evidence‐based resources to establish psychologically safe spaces for conversations with youth with NDD by using a strengths‐based approach to challenge neuro‐normativity and move beyond deficit‐based orientations.

In this study, youth received support from both mental health providers and other types of healthcare providers. Positive mental health conversations were reported when youth felt there was a safe space for disclosure, had their experiences understood and validated, and did not feel rushed. These processes built trust between the youth, their caregiver and HCP and prompted the youth to engage with the support offered by the HCP. While there were instances where a specialist mental health provider was best positioned to provide support (suicidality), study findings identify a collective mental health opportunity for HCPs as youth with NDD have frequent encounters with healthcare systems where co‐occurring mental health needs may arise or be identified. Integrating support for co‐occurring mental health across HCPs may have value as non‐specialist HCPs trained to provide early identification, basic mental health intervention and support for mental health have reported improvements in mental health outcomes among general population youth (Fazel et al., 2014; Patel et al., 2018). Although limited research has examined whether a collective HCP model to mental health improves outcomes for youth with NDD, embedding mental health awareness and capacity across HCP roles could be particularly impactful to support youth with NDD due to narrow role definitions, health systems issues, or resource constraints. Study findings underscore the potential value of proactive, preventative, system‐wide approaches that approach co‐occurring mental health as an essential component of all care, with referrals to specialist care where needed. This was critical as the need for support arose when co‐occurring mental health may not have been the presenting problem. Given that community HCP practitioners report limited confidence in supporting youth and adults with NDD (Maddox et al., 2020), a co‐created Interprofessional Education (IPE) resource “Mental health is everyone's business!” has been developed to enhance compassionate healthcare for youth with NDD across HCPs. Drawing on the digital stories, the IPE curriculum (Mental health is everyone's business! ‐ facilitator's guide) seeks to raise awareness about the mental health needs of youth with NDD and work toward more collaborative approaches to care across HCPs. Research evaluating the impact of the IPE curriculum on supporting mental health conversations across HCPs is warranted.

To support conversations, trust, personalization and rapport‐building were critical components. Fulfilling the second aim of the study, youth highlighted clinical discussions have significant impacts within and beyond the clinical encounter. Relational aspects such as compassion, rapport and trust facilitated supportive mental health conversations. As illustrated in this study, trust was formed and maintained when youth were included in their care plan (involved caregiver if appropriate), were not rushed and provided with reassurance, were seen in more private spaces rather than open concept spaces, felt understood and valued, and received consistency in their care. Communicating with youth in clear and concise ways which suited their needs was consistent with our findings as effective communication can improve care engagement (Moreno‐Durate et al., 2024) and contribute toward positive health outcomes (Weitzman et al., 2024). This is important as youth with NDD are at greater likelihood to experience discrimination, poor healthcare and unmet needs (Menzes et al., 2021). This agential approach employed by HCPs was foundational to build trust and deliver supportive conversations about co‐occurring mental health challenges for youth with NDD and general population youth seeking support in emergency services for a mental health crisis (Bergen et al., 2022). Conversely, trust was undermined when youth felt unseen, judged, invalidated and when power differentials were invoked by HCPs. This breakdown of trust has also been observed among marginalized youth in the general population where trust was undermined and damaged if youth did not feel genuinely cared for and had their agency disrespected by HCPs (Sapiro, 2020). The breakdown of trust can contribute to guardedness in the treatment relationship (Sapiro, 2020), highlighting a peril in vulnerability and disclosure. Findings of this study underscore the importance of trust as a component to build rapport for youth with NDD as good rapport can reduce anxiety, improve adherence to treatment plans (Drmic et al., 2017), whereas challenges with trust can contribute to emotional distress, reduced participation, and ineffective interventions (LaPoint et al., 2024). Intentional, compassionate and consistent approaches which are person‐centered when discussing mental health may be well suited for youth with NDD and warrant further exploration.

The role of family in supporting youth with NDD is both important and complex. Family members were central figures in their support, often serving as the primary and consistent source of support across clinical and everyday contexts. This is consistent with Moses (2010) who emphasizes that families often act as default caregivers and advocates, especially when healthcare support falls short. Involving and supporting families more intentionally in care is not only compassionate, but an essential component for youth with NDD. Engaging parents/caregivers more directly has the potential to better support youth with NDD, given that they are a significant source of support beyond the clinical encounter. While it is important to involve families as partners in care, it is also important to respect the autonomy and voice of youth in as families may not always be a safe or supportive mechanism for all youth. Similar to Moses (2010), family relationships can potentially be a source of distress for youth. Findings of our study highlight the importance of shared decision‐making and family‐centered care in mental health discussions where family members can provide valuable collateral information as essential partners in care, while also respecting the autonomy and voice of the youth. The use of a holistic transprofessional approach (Salimen, et al., 2025) may be of value where youth and family are placed at the center of care issues, and HCPs work in tandem to achieve shared goals, mutual trust and establishing respect.

## STRENGTHS AND LIMITATIONS

The use of co‐production and DST are key strengths of the study. Co‐producing research with youth and parents ensured that the study represented the daily lives, strengths and challenges of receiving co‐occurring mental health support. The selection of DST also was critical as it encouraged the team to choose a methodology that could optimally support the diverse needs, strengths, challenges and abilities of youth with NDD. We selected DST to provide youth with a medium to express difficult or distressing experiences (Jachyra et al., 2024) and to prioritize their voices and perspectives (Jachyra et al., 2019). In this way, DST sought to mobilize their individual strengths and support them to communicate in ways that suited them best. For example, one participant communicated with augmented and alternative communication and typed responses, during virtual group meetings and the digital story, another participant wrote a poem. Two participants initially communicated through drawings and used the drawings to work through their thoughts and experiences. With research suggesting that youth with NDD can experience communication differences (Ciarmoli et al., 2023), DST offered multiple communication modalities. The flexible aspects of DST not only embodied inclusive research, but the accessibility of DST enabled youth voice, choice and empowerment of individual needs to generate in‐depth and nuanced digital stories. The accessibility of DST was well received as youth shared, they felt empowered to communicate authentically on their own terms, and at their own pace and time as they were not under social or time pressures associated with traditional methods such as interviews, surveys or quantitative designs. Finally, the workshop format of DST provided youth with a structured space and place across 24.5 h of engagement to create a sense of community, peer support and friendships among youth. The iterative nature of DST also allowed youth to engage with their peers on a weekly basis to build a safe space as youth shared that they looked forward to seeing their peers at each workshop, encouraged and supported one another during the workshops and developed friendships during and after workshops concluded. Engaging in DST showed six youth that they are not alone in experiencing these challenges, that they deserved to feel safe, seen and heard through high‐quality care, and that hope and recovery are possible. These benefits of DST have been noted in previous mental health research such that engaging in DST can contribute to therapeutic catharsis where connections with others are made, stories are reclaimed, emotionally fraught memories are released (Ferrari et al., 2015). As an interactive and dynamic activity, DST allowed us to journey with participants across a 7‐week period to share vulnerable aspects of their lives and generate a thick description of mental health challenges and experiences seeking support. The cathartic benefit and journeying with participants may have been missed if singular and static approaches were used such as a “drive by” interview (Smith et al., 2025) where data is generated across a single time point and across a short period of time. These downstream benefits highlight the social, methodological and empirical value of DST for youth with NDD, particularly in a mental health context.

There are a few limitations which present opportunities for future research. First, the study was conducted in one province, with a need for future research to ensure it reflects local needs and priorities. Second, the research was limited to the perspectives of youth with NDD, with a need for collaborative research across stakeholders to generate further depth and nuance into systems, practices and social structures which better support their mental health. Research examining why challenges with mental health support happen and examining how mental health prevention, intervention and social issues could be addressed to better support mental health is integral to support the lives and flourishing of youth with NDD. Third, the sample was limited to nine individuals. Despite significant interest in the study, the time intensive nature of the project limited recruitment. Future research with a diversity of participants across NDDs, age and communication abilities would also be beneficial to increase the reach of co‐occurring mental health support.

## CONCLUSION

In this study, we examined the perspectives of youth with NDD about their experiences with receiving mental health support and fostering discussions about co‐occurring mental health with HCPs. Youth highlighted the impacts of negative interactions and the importance of compassionate and relational care, tailored to their needs, communication abilities and comprehension level. Youth also shared specific approaches that would foster positive interactions during encounters with HCPs. Their perspectives and experiences generated strategies, a clinical practice tool and IPE curriculum to better support HCPs. Broadening support to include all HCPs for co‐occurring mental health warrants further examination.

### Clinical resources

Clinician Toolkit: Supporting The Mental Health of Youth Health of Youth with Neurodevelopmental Disabilities: https://pond‐network.s3.amazonaws.com/uploads/ClinicianToolkit‐07.21.2025.pdf.

Interprofessional Education Toolkit: https://hollandbloorview.ca/sites/default/files/2025‐07/Digital‐Stories‐IPE‐Facilitators‐Guide‐July‐2025.pdf.

Digital Stories by Youth: https://braininstitute.ca/resources/brain‐health‐initiatives/pond‐youth‐digital‐stories.

## AUTHOR CONTRIBUTIONS

**Patrick Jachyra**: Investigation; funding acquisition; writing—original draft; writing—review and editing; methodology; formal analysis; resources; project administration; conceptualization; supervision; data curation. **Yona Lunsky**: Conceptualization; funding acquisition; writing—original draft; writing—review and editing; project administration; resources; methodology; formal analysis; supervision; investigation. **Andrada Cretu**: Writing—original draft; writing—review and editing; formal analysis; data curation. **Elim Chan**: Writing—original draft; writing—review and editing; formal analysis; data curation. **Noah Barnett**: Methodology; conceptualization; funding acquisition; writing—original draft; writing—review and editing; formal analysis. **Michael Barnett**: Formal analysis; writing—review and editing; writing—original draft. **Sheldon Gaboury**: Conceptualization; funding acquisition; writing—original draft; writing—review and editing; methodology; formal analysis. **Cathy Gaboury**: Conceptualization; writing—original draft; funding acquisition; methodology; formal analysis; resources; writing—review and editing; supervision; investigation; project administration. **Austin Cosgrove**: Conceptualization; methodology; writing—review and editing; writing—original draft; funding acquisition; formal analysis; data curation. **Susan Cosgrove**: Conceptualization; methodology; writing—review and editing; writing—original draft; funding acquisition; formal analysis; data curation. **Claudine Aquino‐Evangelista**: Conceptualization; funding acquisition; writing—original draft; writing—review and editing; methodology; formal analysis; data curation. **Carla Southward**: Writing—original draft; writing—review and editing. **Evdokia Anagnostou**: Conceptualization; investigation; funding acquisition; writing—original draft; writing—review and editing; methodology; formal analysis; project administration; supervision; data curation.

## CONFLICT OF INTEREST STATEMENT

Dr. E.A. has received grants from Roche, MapLight Therapeutics, and Anavex, served as a consultant to Roche, Quadrant Therapeutics, Cell El, Ono, Acadia Pharmaceuticals Inc., and Impel Pharmaceuticals, has received in‐kind support from AMO Pharma and CRA‐Simons Foundation, received royalties from APPI and Springer, received an editorial honorarium from Wiley, and has a patent for holly™ (formerly Anxiety Meter), with royalties paid from Awake Labs. The remaining authors have declared that they have no competing or potential conflicts of interest.

## ETHICAL CONSIDERATIONS

Informed consent was obtained from all study participants prior to study participation. The study was approved by the Research Ethics Committee at the following hospitals and research institutes: (1) Holland Bloorview Kids Rehabilitation Hospital, REB 0330‐2021, Approval Date: January 27, 2021. (2) Centre for Addiction and Mental Health, REB 133‐2021, Approval Date: January 21, 2021.

## Supporting information

Table S1

## Data Availability

The data that support the findings of this study are openly available in Ontario Brain Institute at https://braininstitute.ca/resources/brain‐health‐initiatives/pond‐youth‐digital‐stories.

## ENDNOTE

^1^
In this paper, the authors use the term neurodevelopmental disorders as this was the term used by youth and families in prior work in the Province of Ontario Neurodevelopmental Disorders Network. Importantly, the authors recognize there are a diversity of perspectives and preferences regarding language use about neurodivergence. They recognize the importance of language and that clinical terminology is fluid and ever‐changing, with the need to revise language on an on‐going basis.
