# Supplementary material for: Supporting mental health conversations among youth with neurodevelopmental disorders through digital storytelling: A co‐produced study
Source: JCPP Adv. 2026 Oct 8:e70179. Online ahead of print. doi: 10.1002/jcv2.70179 (PMC13650193; doi:10.1002/jcv2.70179)
Supplement: Supplementary file 1 [file JCV2-9999-e70179-s001.docx]

**Supporting mental health conversations among youth with neurodevelopmental disorders through**

**digital storytelling: A co-produced study**

**Supporting Information**

**Table S1.**

GRIPP2 short form

| Section and topic | Item | Reported on page No |
| --- | --- | --- |
| 1: Aim  Report the aim of PPI in the study | To work collaboratively with youth diagnosed with neurodevelopmental disorders (NDD) and parents, to ensure that the research conducted meaningfully reflects the daily lives, needs and abilities of youth with NDD and those who care for them. | 6 |
| 2: Methods  Provide a clear description of the methods used for PPI in the study | Youth and parent members were integral team members developing study design, grant writing, data analysis, manuscript writing. They initially conceived the idea for this study and rightly are authors of this paper.  Three youth and four parents volunteered to co-produce the study to form the study team. Youth members all had at least one NDD diagnosis, and parent members all cared for a child diagnosed with an NDD. An equitable approach to co-production was taken where youth and parents were equal partners with planning, power and had decision-making responsibilities (Tan et al., 2025).  From the outset of the study, the team recognized the presence and impact of visible and invisible power dynamics and sought to work through these through open and on-going discussions about power to create a safe space. The team sought to build rapport through regular meetings, empowerment through valuing differing perspectives and providing voice, choice and decision making, particularly for youth team members. | 7 |
| 3: Study results  Outcomes—Report the results of PPI in the study, including both positive and negative outcomes | Throughout analysis, the team engaged reflexively using iterative data analysis, group discussions and peer debriefing led by the first author who had extensive experience conducting qualitative research and co-production among youth with NDD. Engaging in reflexivity was critical as our insider roles were a key resource to develop an in-depth understanding of mental health experiences among youth with NDD.  Concomitantly, it was important to reflect how our identities, life experiences, researcher and clinical training shaped analytic choices as our team included experts through experience from the youth and parents, clinicians, a knowledge translation specialist and researchers with expertise in digital storytelling, psychology, mental health, pediatric medicine, qualitative research and co-production. | 11-12 |
| 4: Discussion and conclusions  Outcomes—Comment on the extent to which PPI influenced the study overall. Describe positive and negative effects | The youth and parent members were central team members and played a pivotal role in discussing the implications of the results that were generated. In the discussion section, they raised the importance of ensuring this knowledge makes it to healthcare professionals (HCPs) on the front line. As such, with the digital stories, a toolkit for HCPs was developed and was linked in the discussion section. This linkage occurred as a result of the contributions of the youth and parents.  The youth and parent members also were integral and helping to write about the collective role for HCPs and the sensitivities that can occur when involving parents and family members in mental health support encounters. The nuance of thinking about including family members and acknowledging that this can also at times be problematic was integral to the message and writing that occurred in the paper, with a role in helping to feedbacking on these sections along with discussion as a whole. | 20-22 |
| 5: Reflections/critical perspective  Comment critically on the study, reflecting on the things that went well and those that did not, so others can learn from this experience | The use of co-production and DST are key strengths of the study. Co-producing this study with youth and parents ensured that the study represented the daily lives, strengths and challenges of receiving co-occurring mental health support and broader. The selection of DST by the youth and parents was also critical as it encouraged the team to choose a methodology that could optimally support the diverse needs, strengths, challenges and abilities of youth with NDD.  This was a unique group, where genuine relationships were formed. It was incredible to see how all team members grew individually within and beyond their comfort zones. We were able to establish a safe space that allowed us to be bold and brave and safe, when thinking about difficult and sensitive issues closely connected to our daily lives through work, life or caregiving. This was one of the most positive experiences of co-production which will be remembered and deserves to be celebrated. | 23 |

PPI=patient and public involvement
